# Self-efficacy of psychiatric nurses and its influencing factors in Guangdong China: A cross-sectional study

**DOI:** 10.1097/MD.0000000000050577

**Published:** 2026-09-18

**Authors:** Dabing Dai, Wenjuan Zhou, Yu Xu, Aixiang Xiao, Yanheng Wei, Junrong Ye

**Affiliations:** a Department of Critical Care Medicine, West China Hospital of Sichuan University, Chengdu, China; b Department of Ophthalmology, West China Hospital of Sichuan University, Chengdu, China; c Department of Nursing Administration, Affiliated Brain Hospital of Guangzhou Medical University, Guangzhou, China.

**Keywords:** cross-sectional study, influencing factors, psychiatric nurses, self-efficacy

## Abstract

Self-efficacy is a critical factor influencing quality of care, particularly for psychiatric nurses at higher occupational risk; however, research on its current status and influencing factors among the nursing population in China remains scarce. This study aimed to investigate the self-efficacy level of psychiatric nurses in Guangdong Province and to analyze its influencing factors. This cross-sectional study utilized convenience sampling to survey psychiatric nurses from 27 hospitals in Guangdong Province, China, between January and May 2022. Self-efficacy was assessed using a validated General Self-efficacy Scale. Univariate and linear regression analyses were used to explore factors influencing self-efficacy among psychiatric nurses. The General Self-efficacy Scale score of the 668 participants was 25.25 ± 5.90. When comparing the self-efficacy scores of psychiatric nurses of different ages, genders, working years, positions, number of night shifts, and whether they were assisted by unlicensed assistive personnel, the differences were statistically significant (*P* < .05). Age, gender, and the presence of unlicensed assistive personnel assistance were influencing factors of self-efficacy (*P* < .001). The study’s findings are consistent with theoretical derivations from Bandura’s Social Cognitive Theory, demonstrating that higher self-efficacy was associated with advanced age, male sex, and unlicensed assistive personnel support. Therefore, targeted interventions focusing on female and junior nurses, including team structure optimization and specific training, are suggested to improve collective self-efficacy among psychiatric nurses.

## 1. Introduction

In China’s mental health service system, psychiatric nurses are at the forefront of managing high-risk clinical scenarios, including responding to violent assaults, intervening in non-suicidal self-injury, and caring for long-term hospitalized patients. As a result, they endure unique and persistent occupational stress.^[1,2]^ In this context, self-efficacy – defined as an individual’s confidence in their ability to successfully perform in specific situations – serves not only as a critical predictor of nursing quality and patient safety,^[3]^ but also as an essential psychological resource for maintaining the mental health of psychiatric nurses and safeguarding against burnout.^[4]^ However, compared with other healthcare professionals, the overall level of self-efficacy among nurses tends to be relatively low.^[5]^ Within the particularly high-risk and emotionally demanding field of psychiatry, this vulnerability may be further amplified, potentially leading to a decline in nursing performance, increased psychological distress, and elevated turnover intention.^[6]^

Self-efficacy is rooted in 4 identifiable sources of information.^[7]^ For psychiatric nurses, these sources are deeply embedded in their daily clinical practice. Understanding how these mechanisms function is a theoretical prerequisite for developing effective intervention strategies.^[8]^

Enactive mastery experience is widely regarded as the most powerful source of efficacy information. For psychiatric nurses, successfully managing a violent incident or effectively calming a patient engaging in self-injury can directly and profoundly reinforce their professional belief in their ability to cope. Conversely, repeated failures in crisis intervention or feelings of helplessness in emergency situations can progressively undermine their sense of efficacy.^[9]^ Research has shown that, in the acquisition of clinical skills, successful hands-on experience plays a decisive role in shaping nurses’ confidence in managing complex situations in the future.^[5]^ Such success derived from front-line practice forms the most solid foundation for the self-efficacy of psychiatric nurses.^[9]^

Vicarious experience operates through observing the performance of peers. Psychiatric nursing is highly contextual and rich in tacit knowledge. When nurses observe colleagues of similar ability and experience successfully managing crisis events or resolving nurse–patient conflicts, they develop an expectancy that “if they can do it, so can I.”^[10]^ Clinical demonstrations during supervision, experience sharing in case discussions, and observing how seasoned nurses handle emergencies with composure all serve as important sources of vicarious experience.^[10]^ Studies indicate that during clinical skills training, observing a peer’s successful performance significantly enhances the observer’s confidence in performing the same task.^[4]^ Such observational learning is particularly vital for early-career psychiatric nurses.^[4]^

Verbal persuasion refers to positive feedback, encouragement, and constructive evaluation from supervisors, colleagues, or the multidisciplinary team. The outcomes of psychiatric nursing are often intangible – patients experience relapses, hospitalizations are prolonged, and nurses’ professional contributions are not always readily visible.^[10]^ Under such circumstances, specific, evidence-based affirmation can help nurses recognize their professional value and reinforce their confidence to persist in challenging situations.^[1]^ Generic praise not grounded in clinical reality has limited effect, whereas authentic feedback based on professional observation can substantially support nurses’ efficacy beliefs.^[5]^

Interpretation of physiological and affective states is particularly relevant to psychiatric nurses. Facing potential violence, unexpected self-injurious behavior, or maintaining prolonged vigilance, nurses frequently experience persistent psychological tension and physiological arousal (e.g., palpitations, insomnia, fatigue). If these reactions are interpreted as signs of incompetence, self-efficacy may be impaired. If, however, they are reappraised as normal responses under stress and managed through emotional regulation to restore a sense of control, such interpretations can help sustain efficacy beliefs.^[10]^ Research suggests that in high-pressure situations, cognitive reappraisal – reframing anxiety as mobilizable energy – can significantly improve both self-efficacy and actual performance.^[4]^ Thus, how nurses perceive and interpret their own physical and emotional responses in crisis situations is itself an integral part of efficacy formation.^[11]^

Currently, empirical research on the current state and influencing factors of self-efficacy among psychiatric nurses in China remains relatively limited.^[9]^ Moreover, the unique local context limits the direct applicability of international findings.^[12]^ First, mental health resources in China are unevenly distributed, and the hierarchical diagnosis and treatment system is still evolving. In Guangdong Province, the setting of this study, the demand for mental health services is substantial due to its large population. Psychiatric nurses in grassroots and underserved areas often face higher workloads and more complex cases while lacking adequate professional support and continuing education opportunities. This restricts their access to positive mastery experiences and effective verbal persuasion.^[13]^ Second, collectivist culture and traditional gender role expectations intersect in complex ways. The nursing workforce is predominantly female, and many nurses must balance intense clinical demands with traditional family role expectations. This role conflict exacerbates emotional exhaustion, thereby affecting self-efficacy.^[14,15]^ Furthermore, safety culture at the hospital and ward levels – including mechanisms for reporting and managing violent incidents, managerial support for nurses’ psychological well-being, and the climate of trust within teams – profoundly influences nurses’ perceptions of their physiological and emotional states and their sense of control over the work environment.^[16]^ A supportive organizational climate can buffer the erosive effects of occupational stress on self-efficacy, whereas an unsupportive one may foster a vicious cycle of stress, inefficacy, and withdrawal.^[17]^

Despite these challenges, there are clear pathways for enhancing self-efficacy among psychiatric nurses. Research has shown that work engagement mediates the relationship between self-efficacy and life satisfaction,^[10]^ and that resources such as psychological capital and perceived organizational support can effectively mitigate burnout.^[18]^ These findings suggest that optimizing management practices, building supportive work environments, and designing training programs based on the 4 sources of efficacy – such as crisis simulations to strengthen mastery experiences,^[19]^ case supervision and peer observation to provide vicarious experiences, positive feedback mechanisms to implement verbal persuasion,^[10]^ and mindfulness and emotional regulation training to improve the interpretation of physiological and affective states^[20]^ – hold promise for systematically enhancing psychiatric nurses’ efficacy beliefs.

In summary, given the increasing burden of mental illness in China and the relative shortage of psychiatric nursing human resources,^[1]^ it is both theoretically important and practically necessary to systematically investigate the level and influencing factors of self-efficacy among psychiatric nurses. Such investigation should be grounded in the theoretical sources of self-efficacy and closely consider China’s unique healthcare system, sociocultural context, and organizational environment. The findings will not only contribute to theoretical understanding but also provide critical evidence for developing culturally tailored interventions aimed at improving nurses’ occupational mental health and quality of care.^[15]^ The present study aims to address this gap by examining the level of self-efficacy among psychiatric nurses in Guangdong Province and analyzing its associated factors, with the goal of informing strategies to enhance self-efficacy in this population.

## 2. Materials and methods

### 2.1. Study design

This cross-sectional study used convenience sampling to survey psychiatric nurses in Guangdong Province, China, who met the inclusion criteria between January 2022 and May 2022. The sample size was calculated according to the following formula: n = 1 + *m* + *m*ψ^2^(1/*R*^2^ − 1) for the study of influencing factors, where m is the number of independent variables, 7. When the 2-sided test α=0.05 is standard, ψ = 1.96. According to previous studies, *R*^2^ = 0.22 was found, and n = 537 was calculated. Considering a 10% loss to follow-up rate, the minimum required sample size was 591. This study included a total of 668 participants.

### 2.2. Ethical consideration

Prior to this study, ethical approval was obtained from the Institutional Review Board (IRB) of the Affiliated Brain Hospital of Guangzhou Medical University (approval number: A2018440). In this study, an online platform was used to collect the data (https://www.wjx.cn). Their background, purpose, inclusion criteria, and informed consent were obtained before completing the questionnaire. If the respondents agreed to participate and clicked the corresponding button, the platform activated the questionnaire automatically.

### 2.3. Participants

The inclusion criteria for participants were as follows: registered nurses, working in a psychiatric hospital or ward for more than 1 year, and giving informed consent to participate in this study. The exclusion criteria were as follows: nursing students, training nurses, nurses who declined to participate or were unwilling to provide informed consent, and nurses not on active clinical duty.

### 2.4. Data collection

The questionnaire was transferred to an online survey platform with a permanent link. Second, the link was sent to the WeChat group of the Psychiatric Nursing Association (PNA) and Mental Health Commission (MHC) of Guangdong Province, and PNA/MHC members were invited to participate in and share this survey with their colleagues. To optimize the response rate, the link was sent once per week during the study period. The instructions for filling out this survey were introduced before oral informed consent was obtained, and participants were asked to complete this survey. The study results were not disseminated to participants. To eliminate repeated submissions, participants were required to link their personal WeChat account (a social application) to an online survey platform before writing the questionnaire, and 1 WeChat account was allowed to submit once. Each questionnaire was screened by the researchers. Questionnaires with poor rationality were excluded after careful discussion with the 3 researchers. A total of 700 questionnaires were distributed. Of these, 693 responses were returned, yielding a preliminary response rate of 99.0%. The returned questionnaires were first screened for validity; 11 responses (1.57%) were excluded because they failed to meet the inclusion criteria (e.g., respondents under 18 years of age or those with implausible work experience greater than their age). Subsequently, an additional 14 responses (2.0%) were discarded because they exhibited straight-line responses (i.e., selecting the same rating for all items). After this data cleaning process, 668 valid questionnaires were retained for analysis, resulting in an effective response rate of 95.43%.

### 2.5. Measurement

#### 2.5.1. General information

The characteristics of the participants were collected, including basic information (gender, age, marital status), current position (clinical care, nursing management, and nursing research and teaching), level of education, job title, time working at psychiatric hospitals, and night shifts. In addition, the environmental factors of the institutes were also recorded, including the acknowledged degree, administrating model, nurse-to-patient ratio, availability of unlicensed assistive personnel (UAP) assistance, and allocation of primary care providers.

#### 2.5.2. Self-efficacy

Self-efficacy was assessed using the General Self-efficacy Scale. This scale was first compiled by Schwarzer^[21]^ and the Chinese version was translated by Wang.^[22]^ The overall Cronbach’s α coefficient of the Chinese version of the scale was 0.87, and the test–retest reliability was 0.83. The scale is unidimensional, with a total of 10 items, and is divided into a 4-point Likert scale: 1 = strongly disagree, 2 = disagree, 3 = agree, and 4 = strongly agree, with a total score of 10 to 40 points. Higher scores indicated greater nurse self-efficacy. In this study, Cronbach’s α for General Self-Efficacy Scale was 0.91.

### 2.6. Statistical methods

Data were analyzed using the Statistical Package for the Social Sciences (SPSS) software (version 22.0; SPSS Inc., Chicago). The measurement data in this study conformed to a normal distribution and are described as the mean ± standard deviation. The independent sample *t*-test and analysis of variance were used for comparisons between the groups. Multiple linear regression was used to analyze the factors influencing self-efficacy, and statistical significance was set at *P* < .05.

## 3. Results

### 3.1. General information and self-efficacy scores of the research subjects

A total of 668 psychiatric nurses were enrolled in this study. As presented in Table 1, the majority of participants were female (78.89%), worked in front-line clinical positions (91.47%), and a high proportion were required to work night shifts (79.79%). The overall self-efficacy score for all participants was 25.25 ± 5.90, with a mean item score of 2.53 ± 0.59.

**Table 1:** Characteristics of participants.

	N (%)	Mean ± SD
Gender		
Male	141 (21.11)	
Female	527 (78.89)	
Age (yrs)		30.44 ± 7.14
≤25	202 (30.24)	
26–35	301 (45.06)	
36–45	144 (21.56)	
≥46	21 (3.14)	
Marital status		
Unmarried	245 (36.68)	
Married	423 (63.32)	
Education		
Certificate	65 (9.73)	
College	263 (39.37)	
Bachelor	332 (49.70)	
Master	7 (1.20)	
Job title		
Junior	472 (70.7)	
Intermediate	167 (25.0)	
Senior	29 ((4.3)	
Time of working at psychiatric hospitals (year)		8.63 ± 7.23
≤5	275 (41.17)	
6–10	173 (25.90)	
11–15	86 (12.87)	
≥16	134 (20.06)	
Current position		
Clinical care	611 (91.47)	
Nursing management	53 (7.93)	
Nursing research and teaching	4 (0.60)	
Night shift		
Yes	533 (79.79)	
No	135 (20.21)	
Hospital level		
Primary	81 (12.13)	
Secondary	353 (52.84)	
Tertiary	234 (35.03)	
Administrating model		
Secured	504 (75.45)	
Mixed	56 (8.38)	
Non-secured	108 (16.17)	
Nurse-to-patient ratio		0.22 ± 0.12
<0.15	191 ((28.70)	
0.15–0.24	271 (40.70)	
≥0.15	204 (30.60)	
UAP		
Yes	257 (38.47)	
No	411 (61.53)	
GSES		2.52 ± 0.59
1–1.99	104 (15.60)	
2–2.99	416 (62.50)	
≥3	146 (21.90)	

GSES = General Self-Efficacy Scale, Mean ± SD = mean ± standard deviation, N (%) = number (percentage), UAP = unlicensed assistive personnel.

### 3.2. Total score ratio of self-efficacy among psychiatric nurses with different characteristics

Psychiatric nurses in Guangdong were included in information groups based on general information, and their general self-efficacy scores were compared. The results showed statistically significant differences in the total self-efficacy scores of different genders, ages, professional titles, positions, whether they worked night shifts, and whether they had UAP assistance (*P* < .05; see Table 2).

**Table 2:** Total score ratio of self-efficacy among psychiatric nurses with different characteristics.

Item	n	Self-efficacy total score	*Z*	*P*
Gender			3.988*	.000
Male	141	26.99 ± 6.76		
Female	527	24.79 ± 5.57		
Age			6.598†	.000
≤25	202	24.05 ± 5.79		
26–35	301	25.28 ± 5.99		
36–45	144	26.88 ± 5.47		
≥46	21	25.14 ± 6.30		
Job title			3.902†	.021
Junior	472	24.86 ± 6.02		
Intermediate	167	26.06 ± 5.45		
Senior	29	26.93 ± 5.97		
Current position			2.871*	.004
Clinical	611	25.05 ± 5.87		
Nonclinical	57	27.38 ± 5.82		
Night shift			2.179*	.030
Yes	533	25.00 ± 5.97		
No	135	26.24 ± 5.53		
UAP			3.427*	.001
Yes	257	26.23 ± 5.99		
No	411	24.64 ± 5.76		

* *t*-test.

† Analysis of variance.

n = number, *P* = probability value, UAP = unlicensed assistive personnel, *Z* = Z value.

### 3.3. Multiple linear regression analysis of influencing factors of psychiatric nurses’ self-efficacy

The total self-efficacy of this group of psychiatric nurses was used as the dependent variable. The 5 statistically significant items from the single-factor analysis (gender, age, professional title, position, night shift, and UAP assistance) were used as self-efficacy variables, and a multiple linear regression analysis was performed. Categorical variables were dummy-coded, with the following categories designated as the reference category: male, age below 25 years, junior professional title, clinical position, night shift participation, and UAP. The regression model was statistically significant (*F* = 8.924, *P* < .001). However, the low *R*^2^ value of 0.075 (adjusted *R*^2^ = 0.066) indicated that only 7.5% of the variance in the outcome variable could be explained by the included predictors. Collinearity statistics showed that the tolerance range was 0.542 to 0.993 and the variance inflation factor range was 1.007 to 1.845, confirming that there was no multicollinearity in the independent variables. The results of multiple collinear regression analysis showed that age, gender, and UAP assistance were influencing factors of psychiatric nurses’ self-efficacy (*P* < .001; see Table 3).

**Table 3:** Multiple linear regression analysis of influencing factors of psychiatric nurses’ self-efficacy.

Item	B	SE	β	t	P	95%CI
Constant	28.958	1.557	-	18.598	0.000	25.90–32.01
Age	1.137	0.356	0.155	3.190	0.001	0.44–1.84
Gender	−2.439	0.543	-0.169	−4.488	0.000	−3.5 to−1.37
Job title	0.160	0.535	0.015	0.299	0.765	0.89–1.21
Current position	1.461	0.926	0.069	1.577	0.115	−0.36 to 3.28
Night shift	−0.438	0.699	−0.030	−0.627	0.531	−1.81 to 0.93
UAP	−1.772	0.455	−0.146	−3.895	0.000	−2.67 to −0.88

Model statistics: *R*^2^ = 0.075, adjusted *R*^2^ = 0.066, *F* = 8.924, *P* < .001.

β = standardized coefficient, B = unstandardized coefficient, CI = confidence interval, SE = standard error, UAP = unlicensed assistive personnel.

## 4. Discussion

### 4.1. Psychiatric nurses‘ self-efficacy is at a moderate level

This study found that the self-efficacy of psychiatric nurses in Guangdong Province was moderate, with a specific score of 25.25 ± 5.90 and a mean item score of 2.53 ± 0.59. This moderate level is consistent with the widespread phenomenon of occupational stress and psychological distress among psychiatric nurses in China, as reported in current studies.^[4,23]^ From a theoretical standpoint, this moderate level may be conceptually linked to the structural constraints within China’s hierarchical medical system.^[24]^ As a populous province, Guangdong suffers from an uneven distribution of mental health resources, with primary healthcare facilities lacking professional support and continuing education.^[25]^ Meanwhile, patients’ distrust of primary care leads a large number of them to seek services at tertiary hospitals, resulting in excessive workload at these institutions.^[25]^ This bidirectional structural imbalance is likely to limit psychiatric nurses’ opportunities to accumulate positive mastery experiences, such as successfully managing a full spectrum of cases, and to receive effective verbal persuasion, such as constructive feedback and recognition from supervisors.^[26]^ Consequently, the deprivation of these 2 specific sources – mastery experience and verbal persuasion – may partially account for the constrained enhancement of their self-efficacy.

Significant variations were observed in the overall self-efficacy scores among different groups based on gender, age, professional titles, positions, night shift work, and availability of UAP. Nurses without a designated position, holding a junior professional title, or working night shifts exhibited slightly lower levels of self-efficacy than the median scores. When interpreting these subgroup differences through the lens of Bandura’s framework, we propose the following theory-informed associations.^[7]^ First, assuming job responsibilities, such as holding a designated position or senior title, may offer nurses more frequent exposure to complex challenges and skill-development opportunities. The successful resolution of these work-related challenges represents a direct form of mastery experience, which in turn enhances their organizational, communication, and coordination skills, ultimately reinforcing their confidence in problem-solving. Furthermore, night shift work was associated with nurses’ sleep quality and overall health status. Second, the link between night shifts and lower self-efficacy appears to be closely related to the affective and physiological source of Bandura’s theory. Nurses with better sleep quality tend to possess sufficient physical strength to cope effectively with demanding workloads and life pressure. Similarly, nurses with better health status have a more positive outlook on life and work, enabling them to respond adeptly to pressure and swiftly recover from negative emotions. In other words, poor physical condition and fatigue may generate negative affective arousal, which weakens their perceived capability to perform nursing tasks.

### 4.2. Factors influencing self-efficacy of psychiatric nurses

The results of this study show that age, gender, and the presence or absence of UAP are factors that affect the self-efficacy of psychiatric nurses. Given that Bandura’s 4 proposed sources of self-efficacy – mastery experiences, vicarious learning, verbal persuasion, and affective states – were not directly measured in our study, the following interpretations should be understood as theory-informed possibilities rather than empirically verified mechanisms.

#### 4.2.1. Age

This study identified the significant influence of age on psychiatric nurses’ self-efficacy. Specifically, nurses aged 25 years or younger demonstrated the lowest self-efficacy levels among all participants. This observation may be attributed to the typically uncertain career planning and limited work experience of newly employed, younger nurses. In China, many junior nurses enter the field of psychiatric care immediately after completing their university education, without receiving specialized training in this specific discipline.^[27]^ Consequently, their knowledge and understanding of psychiatric nursing practices may have been relatively limited. Theoretically, this lack of preservice training is likely to deprive young nurses of sufficient opportunities to accumulate positive mastery experiences, such as successfully de-escalating aggressive patients or managing prolonged therapeutic relationships, during their initial career phase.^[28]^ Concurrently, the distinctive nature of the psychiatric setting – characterized by higher risks of patient violence and longer average hospital stays – may trigger heightened negative affective arousal, including anxiety and fear, among newly hired young nurses when confronting complex or high-pressure situations.^[29]^ This elevated emotional distress, according to Bandura’s framework, serves as a potent source undermining their perceived capability. Conversely, the relatively higher self-efficacy observed among older nurses may be conceptually associated with the longitudinal accumulation of mastery experiences and the development of effective coping mechanisms over years of on-the-job training.^[30]^ As nurses age and gain seniority, they are more likely to have encountered and successfully resolved a broader array of workplace challenges, which reinforces their confidence through repeated successful performances. Furthermore, occupational maturity may enable older nurses to better regulate negative emotions provoked by workplace stressors, thereby mitigating the debilitating effects of affective arousal on self-efficacy.^[31]^

#### 4.2.2. Gender

This study found that self-efficacy among male psychiatric nurses was significantly higher than among female nurses, consistent with previous research.^[32]^ Drawing on Bandura’s 4 sources of self-efficacy, we offer the following theory-informed interpretations.

First, traditional Chinese gender roles may shape distinct occupational psychological experiences. Men are socially expected to exhibit toughness and emotional stability – traits aligned with the demands of high-risk psychiatric environments – and may therefore perceive crisis situations as controllable “challenges,” facilitating positive mastery experiences.^[33]^ In contrast, women, facing greater societal tolerance for emotional expression, may interpret physiological arousal (e.g., tension, fear) as incompetence signals, potentially inhibiting self-efficacy.^[34]^ However, alternative explanations – such as gender differences in self-report tendencies or workplace dynamics – cannot be ruled out. Second, work-family conflict may exacerbate this gap. Female nurses often bear dual burdens of clinical work and family caregiving due to the traditional division of labor,^[35]^ which may deplete psychological resources and limit access to vicarious experiences and verbal persuasion.^[36]^ Male nurses, facing more singular family role expectations, may have more time for professional development and receive more positive feedback.^[37]^ Third, occupational expectation stress differs by gender. The expectation of men as primary breadwinners may translate into stronger achievement motivation, prompting them to proactively seek promotions and managerial roles, thereby accumulating enactive mastery experiences.^[38]^ Female nurses might forgo such opportunities due to family obligations.^[38]^ Yet organizational factors – such as inflexible scheduling – may also play a role. Fourth, gender differences in safety perceptions may influence self-efficacy. Female nurses reported significantly greater fear of workplace violence and may be more prone to attribute physiological responses (e.g., palpitations, insomnia) to personal inadequacy. Male nurses may engage in cognitive reappraisal to regain control.^[39]^ However, cognitive reappraisal was not assessed, and alternative explanations – including differential violence exposure or organizational support – remain possible.

#### 4.2.3. Unlicensed assistive personnel assistance

This study found that psychiatric nurses working in wards with UAP demonstrated significantly higher self-efficacy. The beneficial effect of UAP support on psychiatric nurses’ self-efficacy may be conceptually linked to multiple sources within Bandura’s framework. First, through the pathway of mastery experiences, UAP assistance with basic nursing tasks may reduce nurses’ routine workload, thereby affording them greater time and energy to focus on specialized professional responsibilities – such as psychological support, patient observation, and treatment implementation.^[40]^ The successful execution of these higher-level clinical tasks may provide nurses with more frequent and meaningful opportunities to accumulate positive mastery experiences, which Bandura identified as the most influential source of self-efficacy. The nurse-to-patient ratio observed in this study (0.22:0.40) is considerably lower than the national standard, suggesting that without UAP support, nurses would face even greater task overload, potentially depriving them of opportunities to engage in and succeed at professionally fulfilling activities.^[40]^

Second, through the affective pathway, workload reduction via UAP assistance may alleviate the negative emotional arousal associated with excessive task demands. Under high workload conditions, nurses commonly experience stress, anxiety, and exhaustion, which Bandura identified as sources of negative affective interpretation that undermine self-efficacy.^[41]^ By performing nonspecialized duties such as physical care, UAP may reduce these stressors, allowing nurses to maintain more positive emotional states and thereby preserve their confidence in handling challenging clinical situations.^[41,42]^

Third, through pathways related to vicarious learning and verbal persuasion, the presence of UAP may facilitate more effective interdisciplinary collaboration. Effective communication among physicians, nurses, and UAP plays a vital role in improving care quality^.[43,44]^ Within such collaborative environments, nurses may have increased opportunities to observe colleagues’ successful practices, representing vicarious learning, and receive constructive feedback from supervisors and team members, representing verbal persuasion, both of which may reinforce their professional self-efficacy. Improved delegation and communication between nurses and UAP can enhance patient care results,^[45]^ and these positive patient outcomes may, in turn, provide nurses with additional mastery experiences. Family involvement is recognized as a protective factor in mental health recovery,^[46]^ and when UAP assist with basic care tasks, they may enable nurses to dedicate more attention to family engagement and psychoeducational interventions. The successful delivery of these family-centered services may represent another domain of mastery experience that contributes to nurses’ overall self-efficacy.

However, alternative explanations for the observed association warrant consideration. First, it is possible that wards with better overall working conditions and more adequate staffing are simultaneously more likely to employ UAP and to have higher nurse self-efficacy, suggesting a confounding effect rather than a direct causal pathway. Second, nurses who already possess higher self-efficacy may be more confident in delegating tasks to UAP and more effective in team coordination, which could result in better utilization of UAP support. Our cross-sectional design cannot distinguish between these competing explanations.

## 5. Conclusion

Based on our empirical findings and Bandura’s framework, we offer the following theory-informed practice implications. For novice junior nurses, whose lower self-efficacy may conceptually relate to insufficient mastery experiences, tiered simulation training could be implemented to facilitate enactive mastery accumulation. For UAP collaboration, which was associated with higher self-efficacy in our sample, standardized workflows and interdisciplinary case discussions may provide observational learning platforms to enhance vicarious experiences. For female nurses facing dual burdens, flexible scheduling and positive feedback mechanisms may alleviate work-family conflict and reinforce verbal persuasion, while mindfulness training might help reframe physiological arousal as manageable responses, potentially improving affective interpretation. Additionally, to address the structural constraints within the hierarchical medical system, it is recommended to facilitate the downward redistribution of quality resources by establishing paired assistance programs between tertiary and primary hospitals and leveraging digital platforms to ensure professional support and simulation training for primary-level nurses. Positive feedback mechanisms should be established to reinforce nurses’ sense of professional value, and self-efficacy-oriented interventions – such as assigning appropriately challenging tasks and encouraging narrative reflection – should be integrated into nursing management to help nurses actively cope with stress.

## 6. Limitations

This study had several limitations that should be acknowledged. First, although Bandura’s 4 sources guided our interpretations, they were not directly measured, making our theoretical discussion inferential rather than empirically validated. Second, the cross-sectional design precludes causal inferences. Third, the sample was limited to Guangdong Province, restricting generalizability. Fourth, the regression model exhibited limited explanatory power (*R*^2^ = 0.075), suggesting that important predictors – particularly those tapping into Bandura’s 4 sources – may have been omitted. Future research should employ longitudinal designs, broader sampling, direct measures of the 4 sources, and mediation analyses to test the proposed pathways.

## Author contributions

**Conceptualization:** Dabing Dai.

**Data curation:** Dabing Dai, Yu Xu, Yanheng Wei.

**Formal analysis:** Dabing Dai.

**Investigation:** Dabing Dai.

**Methodology:** Dabing Dai.

**Resources:** Wenjuan Zhou, Junrong Ye.

**Supervision:** Yu Xu, Aixiang Xiao.

**Validation:** Dabing Dai.

**Writing – original draft:** Dabing Dai, Wenjuan Zhou, Yu Xu.

**Writing – review & editing:** Dabing Dai, Yu Xu.

## References

[1] Wang XX Wang LP Wang QQ. Related factors influencing Chinese psychiatric nurses’ turnover: a cross-sectional study. J Psychiatr Ment Health Nurs. 2022;29:698–708. 10.1111/jpm.12852 35716343

[2] Liu SJ Wang QN She J Zhang YH Xu H. Relationship between emotional intelligence and job stressors of psychiatric nurses: a multi-centre cross-sectional study. J Clin Nurs. 2023;32:7730–9. 10.1111/jocn.16865 37661580

[3] Sahebi Z Barkhordari-Sharifabad M. Spiritual care competency and its relationship with clinical self-efficacy in nursing students. BMC Med Educ. 2023;23:937. 10.1186/s12909-023-04937-3 PMC10709853 38066560

[4] Zhang X Liu L Ning J. The mediating effect of general self-efficacy between occupational stress and negative emotion among psychiatric nurses. J Psychosoc Nurs Ment Health Serv. 2023;61:33–9. 10.3928/02793695-20220809-02 35993726

[5] Van Dyk J Siedlecki SL Fitzpatrick JJ. Frontline nurse managers’ confidence and self-efficacy. J Nurs Manag. 2016;24:533–9. 10.1111/jonm.12355 26762223

[6] Cabrera-Aguilar E Zevallos-Francia M Morales-García M. Resilience and stress as predictors of work engagement: the mediating role of self-efficacy in nurses. Front Psychiatry. 2023;14:1202048. 10.3389/fpsyt.2023.1202048 PMC10464840 37649562

[7] van der Bijl JJ Shortridge-Baggett LM. The theory and measurement of the self-efficacy construct. Sch Inq Nurs Pract. 2001;15:189–207. 11871579

[8] Yada H Odachi R Adachi K. Validity and reliability of psychiatric nurse self-efficacy scales: cross-sectional study. BMJ Open. 2022;12:e055922. 10.1136/bmjopen-2021-055922 PMC8744105 34996799

[9] Yada H Abe H Odachi R Adachi K. Exploration of the factors related to self-efficacy among psychiatric nurses. PLoS One. 2020;15:e0230740. 10.1371/journal.pone.0230740 PMC7117702 32240210

[10] Hussein RM Ali EA El-Ashry AM El-Sayed MM Khedr MA. The power of self-efficacy in fostering caring self-efficacy and overcoming job-related stress and perceived stigma among psychiatric nurses. Int J Nurs Pract. 2026;32:e70157. 10.1111/ijn.70157 42206694

[11] Lee SY Li CY Hsiao PC. Perceived stress and burnout among nurses in acute and critical care settings: the mediating role of self-efficacy. Nurs Crit Care. 2026;31:e70364. 10.1111/nicc.70364 PMC12862118 41622437

[12] Jiang W Yuan Y Zhang L. Self-efficacy and research capacity of clinical nurses in China. J Contin Educ Nurs. 2019;50:509–16. 10.3928/00220124-20191015-07 31644812

[13] Rafiei S Souri S Nejatifar Z Amerzadeh M. The moderating role of self-efficacy in the relationship between occupational stress and mental health issues among nurses. Sci Rep. 2024;14:15913. 10.1038/s41598-024-66357-7 PMC11237126 38987325

[14] Yang SH Tsan YT Hsu WT. Association between self-efficacy, spiritual well-being and the willingness to provide spiritual care among nursing staff in Taiwan: a cross-sectional study. BMC Nurs. 2024;23:299. 10.1186/s12912-024-01978-x PMC11059632 38689216

[15] Wang X Liu M Leung AYM. Nurses’ self-efficacy, job embeddedness, and psychological empowerment: a cross-sectional study. J Nurs Manag. 2025;2025:6259635. 10.1155/jonm/6259635 PMC11999754 40236785

[16] Chen Y He Y Wang P. The association between the adverse event reporting system and burnout and job satisfaction of nurses: workplace violence as a mediator. Int Nurs Rev. 2024;71:1053–61. 10.1111/inr.12962 38650586

[17] Alissa NA Hazazi AM. Mental health nurses’ self-efficacy and quality of professional life toward inpatient violence in mental health hospitals in Saudi Arabia. Perspect Psychiatr Care. 2024;5944058:10.

[18] Hamid R Al-Husban R Alkhawaldeh J. Exploring the relationship between self-efficacy and job performance among nurses: an international perspective. Nurs Manag (Harrow). 2026;33:25–32. 10.7748/nm.2025.e2163 40955040

[19] Dawra AM Shani H. Impact of job burden and risk-taking tendency on self-efficacy among hospital nurses in Bhakkar. Soc Sci Humanity Res Rev. 2025;3:1304.

[20] Sharma K Parihar A Sharma SK Nebhinani N Mudakavi IB. Nomophobia and its impact on mindfulness and self-efficacy among nurses: an analytical cross-sectional study in the institution of national importance, Western India. J Educ Health Promot. 2024;13:184. 10.4103/jehp.jehp_1057_23 PMC11392256 39268430

[21] Schwarzer R Jerusalem M. Generalized Self-Efficacy Scale. En: Weinman J, Wright S, Johnston M, Editores, Measures in health psychology: A user’s portfolio. Causal and control beliefs. editorial NFER-Nelson; 1995:35–37.

[22] Wang CK Hu ZF Liu Y. Reliability and validity of the general self-efficacy scale. Chin J Appl Psychol. 2001;7:37–40.

[23] Wang J Zheng Z Tang Y. Psychological distress and its influencing factors among psychiatric nurses in China: a cross-sectional study. Front Psychiatry. 2022;13:948786. 10.3389/fpsyt.2022.948786 PMC9428287 36061279

[24] Ying L Fitzpatrick JM Philippou J Huang W Rafferty AM. The organisational context of nursing practice in hospitals in China and its relationship with quality of care, and patient and nurse outcomes: a mixed-methods review. J Clin Nurs. 2021;30:3–27. 10.1111/jocn.15486 32890434

[25] Guo L Du X Wu H. Factors associated with patients’ healthcare-seeking behavior and related clinical outcomes under China’s hierarchical healthcare delivery system. Front Public Health. 2024;12:1326272. 10.3389/fpubh.2024.1326272 PMC11047042 38680927

[26] Azemi S Dianat I Abdollahzade F Bazazan A Afshari D. Work-related stress, self-efficacy and mental health of hospital nurses. Work. 2022;72:1007–14. 10.3233/WOR-210264 35634826

[27] Xue B Wang L Jiang Z. Factors influencing decent work among psychiatric nurses in China: a cross-sectional study. BMC Psychiatry. 2024;24:541. 10.1186/s12888-024-05983-x PMC11292887 39085789

[28] Riggs VF Johnson K Bekhet A Woda A Dreifuerst KT. Does practicum experience matter in psychiatric mental health nursing education? Nurs Educ Perspect. 2026;47:154–9. 10.1097/01.NEP.0000000000001490 41944760

[29] Park HJ Hong SH Kim NH Shin SH. Influences of experience of violence and cognitive-emotion regulation strategies on psychiatric nurses’ post-traumatic stress. Healthcare (Basel). 2025;13:3090. 10.3390/healthcare13233090 PMC12692633 41373308

[30] Mello JJ Bell JF Siegel EO Ward DH. Evaluating psychiatric nursing competencies applied to emergency settings: a pilot role delineation study. Int Emerg Nurs. 2016;25:37–42. 10.1016/j.ienj.2015.07.005 26298649

[31] Yada H Kobayashi M Odachi R Yamane T. Factors relating to self-efficacy among psychiatric nurses. J UOEH. 2017;39:229–34. 10.7888/juoeh.39.229 28904274

[32] Verhaeghe S Duprez V Beeckman D Leys J Van Meijel B Van Hecke A. Mental health nurses’ attitudes and perceived self-efficacy toward inpatient aggression: a cross-sectional study of associations with nurse-related characteristics. Perspect Psychiatr Care. 2016;52:12–24. 10.1111/ppc.12097 25495430

[33] Holroyd EA Bond MH Chan HY. Perceptions of sex-role stereotypes, self-concept, and nursing role ideal in Chinese nursing students. J Adv Nurs. 2002;37:294–303. 10.1046/j.1365-2648.2002.02091.x 11851800

[34] Malonda-Vidal E Samper-García P Llorca-Mestre A Muñoz-Navarro R Mestre-Escrivá V. Traditional masculinity and aggression in adolescence: its relationship with emotional processes. Int J Environ Res Public Health. 2021;18:9802. 10.3390/ijerph18189802 PMC8469901 34574731

[35] Yuan L Li Y Yan H. Effects of work-family conflict and anxiety in the relationship between work-related stress and job burnout in Chinese female nurses: a chained mediation modeling analysis. J Affect Disord. 2023;324:309–16. 10.1016/j.jad.2022.12.112 36586602

[36] Robb M. Self-efficacy with application to nursing education: a concept analysis. Nurs Forum. 2012;47:166–72. 10.1111/j.1744-6198.2012.00267.x 22861653

[37] Yang CI Gau ML Shiau SJ Hu WH Shih FJ. Professional career development for male nurses. J Adv Nurs. 2004;48:642–50. 10.1111/j.1365-2648.2004.03252.x 15548255

[38] Kaplan V. Gender sensitive psychiatry and feminist therapy. Cyp Turk J Psychiatry Psychol. 2021;3:211–27.

[39] Maghelal PK Lara JCF Goonetilleke RS Luximon A. Determinants of self-efficacy of driving behavior among young adults in the UAE: impact of gender, culture, and varying environmental conditions in a simulated environment. Heliyon. 2023;9:e13993. 10.1016/j.heliyon.2023.e13993 PMC10006465 36915511

[40] Greenglass ER Burke RJ. Hospital downsizing, individual resources, and occupational stressors in nurses. Anxiety Stress Coping. 2000;13:371–90.

[41] Orne RM Garland D O’Hara M Perfetto L Stielau J. Caught in the cross fire of change: nurses’ experience with unlicensed assistive personnel. Appl Nurs Res. 1998;11:101–10. 10.1016/s0897-1897(98)80092-x 9757609

[42] Sung M Keum E Roh H Song M. The relationship among job overload, self-efficacy, emotional exhaust and turnover intention in clinical nurses. Korean J Occup Health Nurs. 2013;22:130–9.

[43] Ta’an WF Allama F Williams B. The role of organizational culture and communication skills in predicting the quality of nursing care. Appl Nurs Res. 2024;75:151769. 10.1016/j.apnr.2024.151769 38490801

[44] Lancaster G Kolakowsky-Hayner S Kovacich J Greer-Williams N. Interdisciplinary communication and collaboration among physicians, nurses, and unlicensed assistive personnel. J Nurs Scholarsh. 2015;47:275–84. 10.1111/jnu.12130 25801466

[45] Wong KL Chua WL Griffiths P. Teamwork between registered nurses and unlicensed assistive personnel in acute care settings: a scoping review. Int J Nurs Stud Adv. 2025;8:100293. 10.1016/j.ijnsa.2025.100293 PMC11791319 39906753

[46] Saroca K Sargent J. Understanding families as essential in psychiatric practice. Focus (Am Psychiatr Publ). 2022;20:204–9. 10.1176/appi.focus.20210035 PMC10153504 37153130

